# Hypopituitarism and Rathke's cleft cyst in 48,XXYY Syndrome: new insights into sex chromosome aneuploidies

**DOI:** 10.31744/einstein_journal/2025RC1539

**Published:** 2025-11-13

**Authors:** Rafael Loch Batista, Marilena Nakaguma, Leonardo Andrade Gontijo Tavares, Júlio Américo Pereira Batatinha, Mirian Yumie Nishi, Sorahia Domenice, Mario Padula, Berenice Bilharinho Mendonca

**Affiliations:** 1 Universidade de São Paulo Hospital das Clínicas Faculdade de Medicina São Paulo SP Brazil Laboratório de Investigações Médicas (LIM/42), Hospital das Clínicas, Faculdade de Medicina, Universidade de São Paulo, São Paulo, SP, Brazil.; 2 Universidade de São Paulo Hospital das Clínicas Faculdade de Medicina São Paulo SP Brazil Neuroendocrinology Unit, Hospital das Clínicas, Faculdade de Medicina, Universidade de São Paulo, São Paulo, SP, Brazil.; 3 Universidade de São Paulo Hospital das Clínicas Instituto do Câncer do Estado de São Paulo São Paulo SP Brazil Instituto do Câncer do Estado de São Paulo, Hospital das Clínicas, Faculdade de Medicina, Universidade de São Paulo, São Paulo, SP, Brazil.; 4 Universidade de São Paulo Faculdade de Medicina Hospital das Clínicas São Paulo SP Brazil Department of Radiology, Instituto de Radiologia-INRAD, Hospital das Clínicas, Faculdade de Medicina, Universidade de São Paulo, São Paulo, SP, Brazil.

**Keywords:** Klinefelter syndrome, Hypopituitarism, Rathke's cleft cyst, Endocrine system diseases, Sex chromosomes, Aneuploidy

## Abstract

The 48,XXYY syndrome is a rare sex chromosome aneuploidy associated with diverse physical, developmental, and endocrine abnormalities. This case report highlights a 15-year-old male with 48,XXYY syndrome presenting with hypopituitarism and a Rathke's cleft cyst, offering insights into the interplay between genetic syndromes and pituitary dysfunction. The patient exhibited hyperprolactinemia, central hypothyroidism, central hypoadrenalism, and elevated gonadotropin levels. Brain magnetic resonance imaging revealed a cystic lesion within the sella turcica, consistent with a Rathke's cleft cyst. The patient also had autism and severe essential tremor. Physical examination revealed a reduced testicular volume without gynecomastia, and genetic analysis confirmed a 48,XXYY karyotype. Hormone replacement therapy with prednisone and levothyroxine was initiated, resulting in adequate hormonal replacement. Follow-up magnetic resonance imaging demonstrated stability of the pituitary cyst, with no evidence of progression. This case highlights the importance of hormonal evaluation in patients with rare sex chromosome aneuploidies. Routine pituitary hormone evaluation and imaging should be integral to the care of individuals with 48,XXYY syndrome. These findings highlight the value of a multidisciplinary approach that integrates endocrinology, genetics, and neurology to address the complex needs of these patients. The association between 48,XXYY syndrome and Rathke's cleft cyst raises intriguing questions about the potential links between sex chromosome aneuploidies and pituitary abnormalities. This report emphasizes the need for comprehensive endocrine and structural assessments to optimize patient outcomes by contributing to the limited literature on 48,XXYY syndrome.

## INTRODUCTION

The 48,XXYY syndrome is a rare sex chromosome aneuploidy that occurs in approximately 1 in 18,000-40,000 male births.^(1)^ It is characterized by the presence of two extra sex chromosomes, resulting in a total of 48 chromosomes (XXYY). This syndrome represents a unique variant of sex chromosome aneuploidy, with distinct as well as overlapping clinical features when compared with other conditions, such as Klinefelter syndrome (47,XXY) and other types of tetrasomy involving sex chromosomes (*e.g*., 48,XXXX and 48,XXXY).^(2)^

Patients with 48,XXYY syndrome typically present with a constellation of symptoms, including tall stature, intellectual disability, developmental delays, essential tremor, and behavioral problems such as attention deficit hyperactivity disorder and autism spectrum disorder.^(3)^ In addition, these individuals often exhibit hypogonadism, which is characterized by small testes, low testosterone levels, and infertility.^(1)^ The presence of an additional X and Y chromosome exacerbates the clinical manifestations of Klinefelter syndrome, leading to more severe cognitive and behavioral deficits, as well as a higher prevalence of congenital malformations and medical comorbidities.^(4,5)^

Endocrine abnormalities are common in patients with 48,XXYY syndrome, with hypogonadism being a common feature.^(3)^ However, the presence of hypopituitarism, which involves a deficiency in one or more pituitary hormones, may add another layer of complexity to the clinical picture. Hypopituitarism can result from various etiologies, including structural abnormalities such as Rathke's cleft cysts (RCCs) and benign cystic lesions of the pituitary gland, which can compress adjacent pituitary tissue and disrupt hormone production. Rathke's cleft cyst may be asymptomatic but can also cause symptoms, including headaches, visual disturbances, and hypopituitarism.^(6)^ The relationship between RCCs and sex chromosome aneuploidies is not well established; however, the potential of these cysts to contribute to hormonal imbalances in individuals with 48,XXYY syndrome warrants careful consideration.

This case report describes a male patient with 48,XXYY syndrome who presented with an RCC and hypopituitarism. This case highlights the importance of a comprehensive endocrine evaluation in patients with 48,XXYY syndrome, especially when structural abnormalities of the pituitary gland are present. Additionally, we explored the possible role of extra X-chromosome material in contributing to hypopituitarism and the formation of RCCs, providing insights into the molecular mechanisms that may underlie these associations.

## CASE REPORT

A 15-year-old male with a previous diagnosis of 48,XXYY syndrome was referred for endocrine evaluation due to delayed pubertal progression and abnormal hormone levels. His medical history included autism spectrum disorder, marked essential tremor, impulsive behavior, and impaired social interactions. The patient had no history of seizures or visual complaints.

On physical examination, he presented with a height of 188 cm (Z-score: +1.8), weight of 76kg, and a body mass index of 21.5kg/m^2^. Facial dysmorphisms included hypertelorism and a broad nasal bridge. There was no gynecomastia, and his pubertal development was consistent with Tanner stage 5, with bilateral testicular volumes of 15mL. Neurological examination revealed a bilateral postural tremor and mild dysmetria. He was otherwise normocephalic and normotensive, with no clinical signs of hypopituitarism such as hypoglycemia or hypotension.

**Figure 1:**
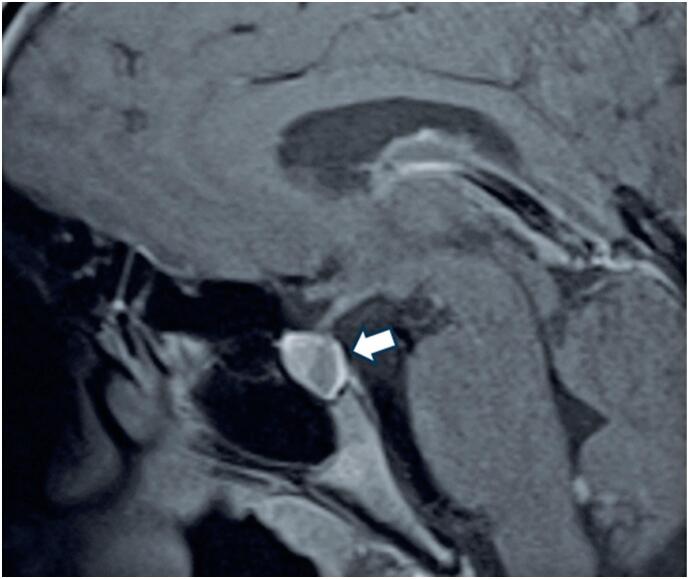
Sagittal post-contrast T1-weighted magnetic resonance imaging showing a non-enhancing, well-circumscribed cystic lesion within the sellar region, consistent with a Rathke's cleft cyst (white arrow). The lesion demonstrates a hyperintense rim with hypointense center, and there is no evidence of suprasellar extension or optic chiasm compression

The patient underwent a cranial computed tomography (CT) scan, which revealed a hypodense lesion in the pituitary region measuring approximately 1 cm in diameter. This finding was confirmed by a subsequent cranial magnetic resonance imaging (MRI) scan, which suggested the presence of an RCC (Figure 1).

Further endocrine evaluation revealed multiple hormonal imbalances, including hyperprolactinemia (prolactin=25.6ng/mL, normal range: 4.2-24.2ng/mL), central hypoadrenalism, hypothyroidism, and elevated gonadotropin levels (Table 1). Karyotype analysis confirmed the 48,XXYY chromosomal pattern. Testicular ultrasound showed reduced testicular volumes (right: 2.5cm³, left: 2.2cm³). The patient began hormone replacement therapy, including prednisone 5mg/day and levothyroxine (75mg/day), to manage endocrine deficiencies.

**Table 1:** Baseline hormonal profile of the 48,XXYY patient

Test	Result	Normal range
ACTH	35.3pg/mL	47.2-63.3pg/mL
DHEA-S	240pg/mL	702-4920pg/mL
Cortisol	5.7µg/dL	6.7-22.6µg/dL
TSH	1.00uUI/mL	0.27-4.20uUI/mL
Free T4	0.88ng/dL	0.93-1.70ng/dL
FSH	29.4IU/L	3.5-12.5IU/L
LH	17.2IU/L	2.4-12.6IU/L
IGF-1	374ng/mL	199-795ng/mL
Prolactin	25.6ng/mL	4.2-24.2ng/mL
Total testosterone	704ng/dL	249-836ng/dL
Free testosterone	375ng/L	131-640ng/L

ACTH: adrenocorticotropic hormone; DHEA-S: dehydroepiandrosterone sulfate; Cortisol: serum cortisol; TSH: thyroid-stimulating hormone; free T4: free thyroxine; FSH: follicle-stimulating hormone; LH: luteinizing hormone; IGF-1: insulin-like growth factor 1; Prolactin: serum prolactin; Total testosterone: Total serum testosterone; Free testosterone: free serum testosterone.

After one year of follow-up, the pituitary cyst remained stable, with no new symptoms. Hormonal assessments indicated adequate pituitary function, with no evidence of additional deficiencies. Surgical intervention was deemed unnecessary, and histopathological examination was not performed.

### Cytogenetic and hormonal analysis

Chromosomal analysis of peripheral blood lymphocytes was performed using standard G-banding at a resolution of 500 bands. A non-mosaic 48,XXYY karyotype was identified in all 20 metaphase cells examined. Hormonal measurements included luteinizing hormone (LH), follicle-stimulating hormone (FSH), total testosterone, and prolactin, all assessed via electrochemiluminescence immunoassays (Roche Diagnostics, Mannheim, Germany). Serum insulin-like growth factor 1 was measured using a chemiluminescent immunometric assay (Siemens Healthcare Diagnostics, Dublin, Ireland). Thyroid-stimulating hormone and free thyroxine levels were measured using an electrochemiluminescence immunoassay (Roche Diagnostics, Mannheim, Germany).

### Ethical approval

This study was conducted in accordance with the principles of the Declaration of Helsinki and was approved by the Ethics Committee for the Analysis of Research Projects of the *Hospital das Clínicas* of the *Faculdade de Medicina* of the *Universidade de São Paulo* (CAAE: 63911317.5.0000.0068; # 1.968.070). Written informed consent was obtained from the patients before the initiation of any research-related procedures.

## DISCUSSION

This case underscores the complex relationship between 48,XXYY syndrome, hypopituitarism, and RCCs, highlighting the challenges in diagnosing and managing these intertwined conditions. As a rare chromosomal anomaly, 48,XXYY syndrome is associated with a spectrum of physical, cognitive, and endocrine abnormalities arising from the presence of additional sex chromosomes. These abnormalities not only complicate the clinical presentation, but also necessitate a nuanced approach to patient management.

Despite presenting with a more severe phenotype, 48,XXYY syndrome shares several phenotypic characteristics with Klinefelter syndrome (47,XXY).^(2)^ In Klinefelter syndrome (KS), certain malignancies occur at higher frequencies, including an increased risk of breast cancer, mediastinal tumors, non-Hodgkin lymphoma, and hematological cancers.^(7)^ Though rare, pituitary gonadotropinomas have been reported in patients with KS. One case involved a 76-year-old man with genetically confirmed KS who developed an oncocytic gonadotrophic macroadenoma.^(8)^ Another case report described a man with long-standing hypogonadism who developed a gonadotropinoma immunoreactive for both FSH and LH.^(8)^ Additionally, a 57-year-old patient with KS was reported to have a pituitary gonadotropinoma, further highlighting the rare but significant association between KS and pituitary adenomas.^(9)^

Rathke's cleft cyst have been reported in aneuploidies of human sex chromosomes. The literature reports a case of a girl with tetrasomy X who developed an RCC, resulting in combined pituitary hormone deficiencies.^(10)^ The similarities between that case and the presentation by our patient suggests a possible shared underlying mechanism linked to chromosomal anomalies. Another notable case involved a 46-year-old Japanese male with a 46,XY/47,XXY karyotype.^(11)^ Hormonal assessments indicated hypogonadotropic hypogonadism, with low serum levels of LH, FSH, and testosterone. Further endocrinological tests revealed hypothalamic panhypopituitarism. Imaging studies, including brain CT and MRI, identified an intrasellar mass. Histological examination after transsphenoidal surgery confirmed the presence of an RCC. Additionally, an incidental RCC was reported in a 22-year-old individual with 48,XXYY having several neurological features but normal hormonal levels.^(12)^

Although RCCs are relatively common incidental findings in adults, they are rare in the pediatric population.^(13)^ Autopsy and imaging studies have shown that the prevalence of RCCs in children is considerably lower than in adults, and when present, the lesions are usually smaller. For example, an MRI study identified asymptomatic pituitary cysts in only 1.2% of children under 15 years of age.^(14)^ The rarity of RCCs in children makes their presence in rare genetic syndromes such as 48,XXYY particularly intriguing and raises the question of whether these occurrences are merely coincidental or reflect shared developmental mechanisms. However, the presence of RCC in our patient, along with other published cases of RCC in individuals with sex chromosome aneuploidies, raises the possibility of shared developmental vulnerabilities. Although definitive causality cannot be established, repeated observations of RCCs in these rare chromosomal syndromes warrant further investigation into potential embryological or genetic links.

Although common, the genetic basis of RCC remains largely unknown. Recently, a rare case of identical twin boys with RCCs was reported, suggesting a likely genetic component in RCC development.^(15)^ In a murine progenitor model, conditional deletion of *Isl1* (*ISL LIM Homeobox 1*) resulted in multiple Rathke's cleft-like cysts with 100% penetrance.^(16)^ Pituitary-specific deletion of *Isl1* leads to hypopituitarism, which is characterized by increased stem cell apoptosis, reduced differentiation of thyrotropes and gonadotropes, and decreased body size.

Notably, individuals with 48,XXYY syndrome possess double the chromosomal material of pseudoautosomal regions (PARs), which are unique segments of sex chromosomes that escape X-chromosome inactivation and are critical for normal development.^(17)^ PARs, located at the distal ends of the X and Y chromosomes, encode several essential genes involved in cell growth, differentiation, and tissue development. These genes are expressed from both sex chromosomes, contributing to the dosage-sensitive regulation of key developmental processes.^(18)^ In individuals with 48,XXYY syndrome, duplication of PARs may result in the overexpression of these genes, potentially leading to developmental abnormalities.

One such gene within the PAR1 family, *SPRY3*, is particularly noteworthy for its role in regulating fibroblast growth factor (FGF) signaling pathways, which are crucial for the development of multiple tissues, including the pituitary gland.^(19)^ Fibroblast growth factor signaling is integral to the early stages of pituitary organogenesis, guiding the proliferation and differentiation of progenitor cells in Rathke's pouch, which is the embryonic precursor to the anterior pituitary.^(20)^ *SPRY3* acts as an inhibitor of this pathway, and its overexpression in 48,XXYY syndrome may lead to the disruption of FGF signaling.^(21)^ This disruption could result in impaired cellular proliferation and differentiation within the Rathke's pouch, contributing to the formation of RCCs.

Although compelling, this proposed mechanism remains hypothetical and is based on indirect evidence from developmental biology and gene expression studies. However, this hypothesis offers a compelling molecular explanation for the pituitary abnormalities observed in sex chromosome aneuploidies and warrants further investigation. Aberrant FGF signaling caused by excessive SPRY3 activity can lead not only to structural anomalies, such as RCCs, but also to functional impairments, such as hypopituitarism. This connection underscores the importance of exploring the roles of *SPRY3* and other PAR genes in pituitary gland development and pathology. Understanding these mechanisms could offer new insights into the broader spectrum of endocrine disorders associated with sex chromosome aneuploidies, paving the way for targeted therapeutic approaches and personalized management strategies for affected individuals. This hypothesis warrants further investigation through genetic and functional studies to elucidate the precise impact of *SPRY3* overexpression on pituitary development and function in 48,XXYY syndrome and related conditions.

## CONCLUSION

This case highlights the importance of comprehensive hormonal evaluation in patients with 48,XXYY syndrome. Routine pituitary imaging and hormonal screening for hypopituitarism should be considered in individuals with chromosomal aneuploidies, including 47,XXY, 48,XXYY, and 48,XXXX. These findings highlight the necessity of a multidisciplinary approach for managing rare genetic syndromes, stressing the importance of early and thorough diagnostic evaluation to identify and address potential endocrine complications. Investigating the potential molecular mechanisms underlying these associations, particularly the role of SPRY3 in pituitary gland development, may provide valuable insights for future studies on congenital hypopituitarism. Additionally, longitudinal studies are warranted to assess the long-term outcomes in patients with 48,XXYY syndrome, which may ultimately refine management strategies and improve patient care.
